# Editorial: Sentinels of the immune system: tissue-resident macrophages in the battle against infection and autoimmunity

**DOI:** 10.3389/fimmu.2025.1759310

**Published:** 2026-01-05

**Authors:** Lalit Batra, Shilpi Giri, Shailesh K. Shahi

**Affiliations:** 1 Center for Predictive Medicine for Biodefense and Emerging Infectious Diseases, University of Louisville, Louisville, KY, United States; 2 Department of Immunology, University of Pittsburgh, Pittsburgh, PA, United States; 3 Department of Pathology, University of Iowa, Iowa City, IA, United States; 4 Graduate Program in Immunology, University of Iowa, Iowa City, IA, United States; 5 Graduate Program in Experimental Pathology, University of Iowa, Iowa City, IA, United States

**Keywords:** autoimmunity, infection, innate immunity, monocyte-derived macrophages (MDMs), tissue-resident macrophages (TRMs)

## Introduction

Macrophages are an integral component of innate immunity and serve as the first line of defense against tissue damage and infection. Tissue-resident macrophages (TRMs) originate from embryonic precursor cells and differentiate into Microglia (brain), alveolar macrophages (lung), Kupffer cells (liver), Langerhans cells (skin), Lamina propria (intestine), red pulp/white pulp/marginal zonal (spleen), peritoneal macrophages (peritoneal cavity) (1). TRMs are seeded into the tissues and maintained throughout lifespan by local self-renewal. TRMs are highly specialized tissue-resident cells with diverse functions and play an important role in maintaining homeostasis by preventing infections, controlling inflammation, and regulating autoimmunity (2).

This Research Topic was conceived to bring together studies that address current challenges and bridge the existing gaps in TRMs biology, including mechanism underlying tissues heterogeneity, interaction between TRMs and other immune cells in controlling pathogenic infections and autoimmune responses, and the development of novel macrophage-targeted therapeutic approaches. The five published articles cover a broad range of topics, including macrophage-mediated immune regulation, disease development, polarization, and emerging therapeutic approaches using both preclinical models and clinical settings. Here, we summarize their key findings, highlighting advances and new insights in TRMs research.

## Role of TRMs in neuroimmune regulation

Xiao and Yang provided an integrated overview of the roles and interactions of tissue-resident macrophages (TRMs) and monocyte-derived macrophages (MDMs) to neuroimmune regulation, particularly within the central nervous system (CNS). The authors outlined the distinct functions of resident versus recruited macrophage populations, demonstrating that resident macrophages (specialized sentinels) primarily maintain homeostasis in the healthy CNS by performing immunosurveillance, clearing debris, and mounting regulated responses to injury. Upon activation, TRMs differentiate into bone marrow-derived macrophages that adopt pro-inflammatory, phagocytic, and antigen-presentation, which may exacerbate or perpetuate neuroinflammation. The review also highlights key mechanism underlaying CNS-macrophage communication. Macrophages express cholinergic receptors such as α7 nAChR and adrenergic receptors, while neurons secrete immunomodulatory proteins (including TAFA4, netrin-1, CART) that influence immune surveillance, tissue repair, regulation of blood-brain barrier permeability, cerebrospinal fluid homeostasis, and overall neuroinflammatory responses.

## Role of TRMs in disease development

Wang et al. elucidated the role of Triggering Receptors Expressed on Myeloid Cells 2 (TREM2). TREM2 receptor is mainly expressed on macrophages, dendritic cells (DCs), myeloid-derived suppressor cells (MDSCs), neutrophils, eosinophils, natural killer (NK) cells, T cells, and mesenchymal stem cells (MSCs). However, the authors primarily focused mainly on TREM2^+^ macrophages, addressing their origin, expression, functions, signaling pathways and further expand on their roles in disease development and potential therapeutic implications. TREM2 triggers NF-κB and MAPK signaling, supporting inflammatory pathways and inducing the expression of pro-inflammatory cytokines (TNF-α, IL-1α, IL-1β) and chemokines (CCL2, CCL3, CX3CR1). Furthermore, expression of these cytokines was significantly reduced in TREM2-KO mice. During respiratory infection such as SARS-CoV-2, TREM2^+^ macrophages express CD163^+^ and MRC1^-^, which protects the lower respiratory tract by producing inflammatory cytokines such as IL-6, TNF, and IL-10. Elevated expression of TREM2 couples with CCL2 production protects pulmonary diseases such as chronic obstructive pulmonary disease, idiopathic pulmonary fibrosis, acute lung injury and tuberculosis. TREM2 is broadly upregulated in tumor-associated macrophages across different tumor types and plays an immunosuppressive role, enabling tumors to evade immune surveillance. It promotes IL-10 production and suppresses T- cells and NK-cell activity, which correlates with poor prognosis. The study also demonstrates that TREM2 enhances the efferocytosis of macrophages and triggers the TREM2-SYK-SMAD4 signaling cascade in cardiovascular disease such as myocardial infarction, sepsis-induced cardiomyopathy, and heart failure which promotes the cardiac function and recovery. In neurological diseases such as Alzheimer’s disease (AD), TREM2 macrophages reduces amyloid-β plaques and maintain microglial survival and function. The authors highlight therapeutics strategies using TREM2+ macrophage agonist antibodies in the treatment of AD, Amyotrophic lateral sclerosis and adult-onset leukoencephalopathy, revealing multiple ongoing clinical trials in these patient populations.

## Impairment of TRMs in ovalbumin (OVA)-induced asthma model

Liu et al. evaluated the ovalbumin (OVA)-induced asthma model to investigate ADAM17-dependent regulation of the scavenger receptor CD36 and further impairment of tissue-resident macrophages (TRMs), particularly alveolar macrophages (AMs). Using flow cytometry and fluorescence assays, the authors quantified TNF-α in bronchoalveolar lavage fluid (BALF), surface expression of ADAM17 and CD36 on AMs, and soluble CD36 (sCD36) concentrations.

The study showed that OVA-induced asthma leads to upregulation of TNF-α and ADAM17 on AMs, which further downregulates CD36 mediated efferocytosis. Based on these findings, the authors concluded that TNF-α inhibitors may serve as promising therapeutic strategy to reduce persistent inflammation in asthma. However, human subject-based validation and long-term functional studies are warranted to establish its translational relevance.

## Macrophage mediated therapeutics against rheumatoid arthritis

Yang et al. investigated the role of various plant-derived metabolites used to treat rheumatoid arthritis (RA). The major pathogenic mechanism in RA involves an imbalance between pro-inflammatory M1 macrophages and anti-inflammatory M2 macrophages (anti-inflammatory), with excessive M1 activity contributing to chronic inflammation. Conventional RA treatments (NSAIDs, DMARDs, biologics) are effective in immunosuppression but often associated with side effects and variable responses. The authors revealed that plant metabolites offer a safe alternative therapeutics option RA treatment. These metabolites either suppress the M1 macrophages (triptolide, glaucocalyxin B, punicalagin, resveratrol, Jolkinolide B, Sappanone A) or promotes M2 macrophages (astragaloside IV, magnoflorin, sinomenine, berberine, naringenin, and luteolin). Additionally, several plant metabolites modulate macrophage polarization *via* metabolic reprogramming and epigenetic regulation. However, most of the provided preclinical data lacks interactions with conventional RA therapies, long-term effects and human trials to test efficacy and safety.

## Macrophage and hypertension research from 2015–2024

Wang et al. conducted a bibliometric and visualization analysis of the literature on macrophages and hypertension research published in last 10 years. Their systemic review revealed macrophage-mediated inflammation as central theme, along with key regulatory mechanisms of hypertension (e.g., angiotensin II pathways), disease comorbidities (atherosclerosis, heart failure, pre-eclampsia), lifestyle factors (diet, high-salt intake), and major molecular players (NLRP3, TNF-α, TGF-β), which emerged as prominent topic clusters. The study utilized clear inclusion criteria, validated bibliometric tools, and a multi-dimensional analytical approach. However, several limitations remain, including reliance on a single database (Web of Science), exclusion of non-English studies, keyword-based clustering, potential citation biases, and lack of differentiation among study types (preclinical, clinical, and reviews). These factors limit the ability to fully assess translational progress in the field.

## Conclusions, future directions, and acknowledgments

In summary, the articles published in this Research Topic advance our understanding of TRMs interaction, function, and their important roles in immune regulation, disease development and therapeutics intervention. Moreover, introduction of innovative approaches such as genomic, transgenic, and advanced immunological technique further expands conceptual, mechanistic, and theoretical knowledge in the TRMs biology. Future directions highlight across these studies emphasize the need for human validation, including well designed clinical trials across diverse disease context, to establish translational relevance and therapeutic potential. We sincerely thank all contributing authors, reviewers, and co-editors for their dedicated efforts and valuable contribution to the successful completion of this Research Topic.
